# Accuracy of Alvarado, Eskelinen, Ohmann, RIPASA and Tzanakis Scores in Diagnosis of Acute Appendicitis; a Cross-sectional Study 

**Published:** 2020-03-13

**Authors:** Mustafa Korkut, Cihan Bedel, Yusuf Karancı, Ali Avcı, Murat Duyan

**Affiliations:** 1 Department of Emergency Medicine, Health Science University Antalya Training and Research Hospital, Antalya, Turkey.; 2 Department of Emergency Medicine, Karaman State Hospital, Karaman, Turkey.

**Keywords:** Appendicitis, Emergency Medicine, Diagnosis, Sensitivity and Specificity, Alvarado, Eskelinen, Ohmann, Raja Isteri Pengiran Anak Saleha Appendicitis, Tzanakis

## Abstract

**Introduction:**

Many scoring systems have been developed to assist in diagnosis of acute appendicitis (AA). This study aimed to compare the screening performance characteristics of Alvarado, Eskelinen, Ohmann, Raja Isteri Pengiran Anak Saleha (RIPASA), and Tzanakis scores in predicting the need for appendectomy in AA patients.

**Methods:**

Our study prospectively evaluated AA patients that were treated in a tertiary hospital’s emergency department. The obtained data were used to calculate Alvarado, Tzanakis, RIPASA, Eskelinen and Ohmann scores. Patients were categorized into two groups according to their histopathological results: positive (PA) and negative appendectomy (NA). The accuracy of different scoring systems in diagnosing AA was investigated.

**Results:**

74 patients suspected to AA with the mean age of 36.68 ± 11.97 years were studied (56.8% male). The diagnosis was histopathologically confirmed in 65 cases (87.8%). Median Alvarado, Tzanakis, RIPASA, Eskelinen and Ohmann scores were significantly higher in patients with positive appendectomy. The area under the curve (AUC), sensitivity, and specificity of Tzanakis score in the cut-off value of 8 were 0.965, 84.4%, and 100%, respectively. For Ohmann and Alvarado scores, these measures were 0.941; 71.9%, 89.9% and 0.938, 60.9%, 89.9%, respectively. Tzanakis scoring system had the best screening performance in detection of cases with AA.

**Conclusion:**

Tzanakis score is more sensitive and specific than Alvarado, RIPASA, Eskelinen and Ohmann scores in identifying AA patients needing appendectomy.

## Introduction

Acute appendicitis (AA) is the most common reason for surgical intervention among patients admitted to the emergency department (ED) with abdominal pain (1). Approximately one-third of AA cases present with atypical clinical symptoms (2). Perforation and negative appendectomy (NA) rates were as high as 12-21% and 13-36%, respectively, for patients that were diagnosed solely through physical examinations (3, 4).

In recent years, many scoring systems have been developed based on anamnesis scores, clinical symptoms and findings, and inflammatory parameters, to assist in diagnosis of AA (5-8). The Alvarado score is the first of these systems. It is based on symptoms, and clinical and laboratory results (9). Then Raja Isteri Pengiran Anak Saleha (RIPASA) system was developed for patients in Asia. In recent years Eskelinen, Ohmann and Tzanakis scores, which added radiological methods such as ultrasound to the scoring systems; clinical and laboratory findings were also followed. These scoring systems aim to reduce NA and mortality/morbidity rates by preventing complications (5-10). 

Despite being inexpensive, reproducible and easy-to-use with high success rates, these systems still have not become a part of routine practice. This study aimed to compare the screening performance characteristics of Alvarado, Eskelinen, Ohmann, RIPASA and Tzanakis scores in predicting the need for appendectomy in AA patients. 

## Methods

***Study design and setting***

In this prospective cross-sectional study, patients who were admitted to the emergency department of a tertiary hospital (Health Science University Antalya Training and Research Hospital, Antalya, Turkey) with abdominal pain suspected to AA between May 2, 2019 and December 1, 2019 were evaluated. This study was approved by the ethics committee of the hospital (Ethics code: 2019-129). All subjects consented to participate in the study, and the data were recorded by ED physicians.

***Participants***

 All cases with abdominal pain suspected to AA, who were referred to ED during the study period, were included using non-probability sampling method. The exclusion criteria were as follows: (a) being under 18 years of age, (b) elective appendectomy, (c) incarcerated or inguinal hernia, (d) non-operable patients, (e) not accepting hospitalization, and (f) incomplete data. 

***Data gathering***

The following data were recorded for all subjects: complaints at the time of admission, and examination and laboratory findings. Significant ultrasonography (US) and abdominal computed tomography (CT) scan findings were also recorded. The following US findings indicated acute appendicitis: (a) non-compressible, (b) >6 mm outer diameter, (c) appendicolith, (d) target appearance in axial section, and (e) periappendiceal inflammation with fat stranding. The following CT findings indicated acute appendicitis: (a) dilated lumen (≥7mm), (b) appendicolith, (c) periappendiceal fluid collection, and (d) inflamed mesoappendix. The obtained data were used to calculate Alvarado, Tzanakis, RIPASA, Eskelinen and Ohmann scores. All patients underwent appendectomy and were categorized into two groups according to histopathologic diagnosis: positive appendectomy (PA) and negative appendectomy (NA). 

***Evaluated Scores***

***Alvarado***

The Alvarado system evaluates 8 parameters, which include symptoms, clinical findings and leukocyte count. The highest possible score is 10, and appendectomy is recommended for scores >7 (11). 

***Ohmann and Eskelinen***

The Ohmann score is also composed of 8 parameters (Tenderness in right lower quadrant, rebound tenderness, presence of urinary system complaint, character of pain, relocalization of pain to the right lower quadrant, age, leukocyte count, abdominal rigidity), a score ≥12 indicates AA (12). In addition to these parameters, the Eskelinen scoring system also considers the duration of pain and laboratory results. A score >57 indicates AA (13). 

***RIPASA and Tzanakis***

Tzanakis et al. developed a scoring system consisting of 4 simplified variables and 15 points based on the combination of clinical evaluation, ultrasonography and laboratory parameters. RIPASA is a scoring system developed for the Asian and middle-eastern population with 15 objective parameters obtained during routine history taking, physical examination, and haematological assessment and urinalysis. A RIPASA score >12 and a Tzanakis score >8 indicate AA (8, 14). 

***Statistical Analysis***

The data were analysed using SPSS version 18.0. Descriptive statistics for categorical data are expressed as numbers and percentages, while mean ± standard deviation and median (minimum-maximum) were used to express continuous data based on normal distribution. Student's t-test was used for variables with normal distribution, and Mann-Whitney U-test was used for variables without normal distribution. The screening performance characteristics of the scoring systems were measured. A greater area under the receiver operating characteristic (ROC) curve (AUC) indicates better diagnostic value. p<0.05 was considered statistically significant.

**Table 1:** Baseline characteristics of studied patients

**Variables**	**Values (n=74)**
**Age (years)**	
Median (min-max)	33 (18-63)
Mean ± standard deviation	36.68 ± 11.97
**Gender, n (%)**	
Male	42 (56.8)
Female	32 (43.2)
**Appendectomy findings for AA**	
Positive	65 (87.8)
Negative	9 (12.2)
**Histopathological findings, n (%)**	
Acute appendicitis	53 (71.6)
Perforated appendicitis	7 (9.4)
Lymphoid hyperplasia	3 (4.0)
Unusual histopathological findings	2 (2.8)
Appendix vermiformis	8 (10.8)
Others	1 (1.4)
**Clinical findings, n (%)**	
Sensitivity on lower right quadrant	64 (86.5)
Defense-rigidity	49 (66.2)
Rebound	44 (59.5)
Fever (>37.3°)	27 (36.5)
Nausea-Vomiting	26 (35.1)
**Laboratory findings**	
WBC count (×10^3^/mm^3^)	14.12±4.71
Neutrophils (×10^3^/mm^3^)	11.10±4.57
Lymphocytes (×10^3^/mm^3^)	1.95±0.82
C-reactive protein (mg/dL)	24 (0-331)
**Scores, median (min-max)**	
Alvarado	7 (2-10)
Ohmann	13 (4-16)
RIPASA	10 (4.5-13.5)
Tzanakis	13 (3-15)
Eskelinen	51.1 (29.8-67.6)

AA: acute appendicitis; WBC: white blood cell; RIPASA: Raja Isteri Pengiran Anak Saleha Appendicitis.

**Table 2:** Comparing the baseline characteristics as well as acute appendicitis scores between cases with positive and negative appendectomy findings

**Variables**	**Appendectomy findings**		**P value**
	**Negative (n=9)**	**Positive (n=65)**	
**Age (years) **			
Median (min-max)	27 (19-46)	36 (18-63)	0.006
**Gender, n (%)**			
Male	3 (33.3)	39 (60)	0.163
Female	6 (66.7)	26 (40)	
**Ultrasonography findings, n (%)**			
Negative	5 (55.6)	28 (43.1)	0.501
Positive	4 (44.4)	37 (56.9)	
**Computed tomography scan findings, n (%)**			
Negative	5 (71.4)	2 (3.6)	<0.001
Positive	2 (28.6)	55 (96.4)	
**Laboratory findings**			
WBC count (×10^3^/mm^3^)	10.38±3.00	14.64±4.69	0.01
Neutrophils (×10^3^/mm^3^)	7.10±2.91	11.66±4.51	0.004
Lymphocytes (×10^3^/mm^3^)	2.60±0.85	1.86±0.78	0.022
C-reactive protein (mg/dL)	9 (0-321)	33 (0-331)	<0.001
**Clinical findings, n (%)**			
Sensitivity on lower right quadrant	3 (33.3)	61 (93.8)	<0.001
Defense guarding	3 (33.3)	46 (70.8)	0.026
Rebound	3 (33.3)	41 (63.1)	0.146
Fever (>37.3°)	4 (44.4)	23 (35.4)	0.716
Nausea-Vomiting	2 (22.2)	24 (36.9)	0.480
**Scores, median (min-max)**			
Alvarado	4 (2-5)	7 (3-10)	<0.001
Ohmann	8 (4-13)	13.5 (8-16)	<0.001
RIPASA	6 (4.5-8)	10 (4.5-13.5)	<0.001
Tzanakis	4 (3-7)	13 (3-15)	<0.001
Eskelinen	35.1 (33.8-49.2)	53.9 (29.8-67.6)	<0.001

WBC: White blood cell; RIPASA: Raja Isteri Pengiran Anak Saleha Appendicitis; min: minimum; max: maximum.

**Table 3:** Screening performance characteristics of different scoring systems in prediction of acute appendicitis in emergency department

	**Alvarado**	**Ohmann**	**RIPASA**	**Tzanakis**	**Eskelinen**
**TP**	40	47	55	56	42
**TN**	8	8	8	8	7
**FP**	1	1	1	1	2
**FN**	25	18	10	9	23
**Sensitivity**	60.9 (48.64-73.35)	71.9 (59.81-82.69)	75 (64.81-86.47)	84.4 (75.34-93.47)	64.1 (51.77-76.08)
**Specificity**	89.9 (51.75-99.72)	89.9 (51.75-99.72)	99.72 (51.75-100)	99.88 (51.75-99.72)	78 (39.99-99.19)
**PPV**	97.56 (86.19-99.61)	97.92 (88.04-99.67)	98.04 (88.69-99.69)	98.25 (89.80-99.72)	95.45 (85.93-98.63)
**NPV**	24.24 (17.89-31.98)	30.77 (21.98-41.21)	34.78 (24.44-46.80)	47.06 (31.72-62.97)	23.33 (15.86-32.96)
**PLR**	5.54 (0.86-35.56)	6.51 (1.02-41.55)	6.92 (1.09-44.15)	7.75 (1.22-49.24)	2.91 (0.85-10.00)
**NLR**	0.43 (0.29-0.64)	0.21 (0.20-0.49)	0.26 (0.16-0.43)	0.16 (0.08-0.30)	0.45 (0.28-0.73)
**AUC**	0.93 (0.87-0.99)	0.94 (0.88-1.00)	0.89 (0.81-0.97)	0.96 (0.90-1.00)	0.86 (0.77-0.97)

Data are presented with 95% confidence interval (CI). Measures are calculated in cut-offs: ≥8 for Alvarado score; ≥12 for Ohmann score; ≥12 for RIPASA score; ≥8 for Tzanakis score; ≥57 for Eskelinen score.

TP: true positive; TN: true negative; FP: false positive; FN: false negative; PPV: positive predictive value; NPV: negative predictive value; PLR: positive likelihood ratio; Negative likelihood ratio; AUC; area under the receiver operating characteristic (ROC) curve.

**Figure 1:**
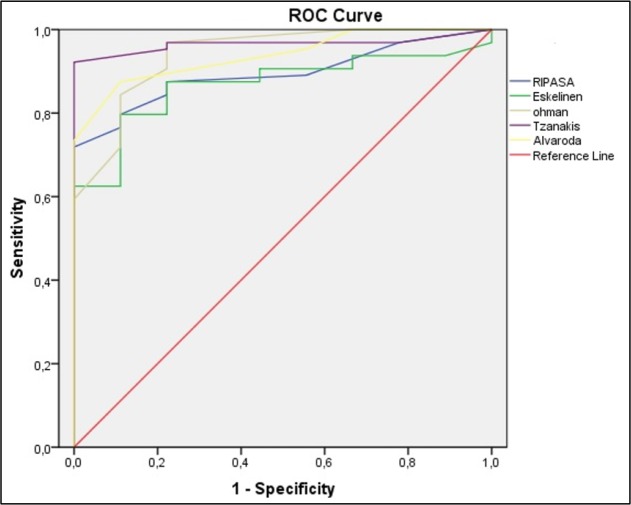
The area under the Receiver operating characteristic (ROC) curve of studied scoring systems in detecting acute appendicitis cases with positive appendectomy in emergency department. (p <0.001 for all scoring systems)

## Results

***Baseline characteristics of studied cases***

The study included a total of 74 patients with a preliminary AA diagnosis: 42 males (56.8%) and 32 females (43.2%). Table 1 shows the baseline characteristics of studied cases. The median age was 33 (range: 18-63) years. The diagnosis was histopathologically confirmed in 65 cases (87.8%). Among these, 7 patients (9.4%) had perforated AA and 3 (4.0%) had lymphoid hyperplasia. 9 (12.2%) patients had negative appendectomy, 1 of these (1.4%) being ovarian cyst rupture. The mean white blood cell (WBC) count was 14.12 ± 4.71 ×10^3^/mm^3^. The median scores of different systems were as follows: Alvarado score 7 (2-10); Ohmann score 13 (4-16); RIPASA score 10 (4.5-13.5); Tzanakis score 13 (3-15); Eskelinen score 51.1 (29.8-67.6).

***Comparing the scores***

Table 2 compares the baseline characteristics as well as scores between cases with negative and positive appendectomy. Median age was significantly higher in patients with positive appendectomy (p=0.006). There was no significant difference between patients with positive and negative appendectomy regarding gender (p=0.163). Ultrasonography results were not sufficient for diagnosing AA (p = 0.501); however, computed tomography (CT) scans were able to significantly determine AA (p <0.001). Median Alvarado, Tzanakis, RIPASA, Eskelinen and Ohmann scores were significantly higher in patients with positive appendectomy. 

Screening performance characteristics of the studied systems in determining cases with AA are presented in table 3 and figure 1. Tzanakis score was able to determine AA better than the other scoring systems, followed by Ohmann and Alvarado scores, respectively (based on AUC). AUC, sensitivity, and specificity of Tzanakis score in the cut-off value of 8 were 0.965, 84.4%, and 100%, respectively. For Ohmann and Alvarado scores, these measures were 0.941; 71.9%, 89.9% and 0.938, 60.9%, 89.9%, respectively. Tzanakis scoring system had the best screening performance in detection of cases with AA.  

## Discussion

Based on the ﬁndings of the present study, Tzanakis score has higher sensitivity and specificity in the diagnosis of AA compared to Alvarado, RIPASA, Eskelinen and Ohmann scores. 

The differential diagnosis of AA only requires simple physical and laboratory analyses; however, it is commonly misdiagnosed due to atypical findings. Perforation and NA rates are still significantly high. The importance of timely and precise diagnosis has led researchers to develop different scoring systems (15). Alvarado is the first and most widely used among them (10). It is simple, easy-to-use and can successfully predict AA (16). Subraman et al. reported the sensitivity and specificity of Alvarado score to be 68% and 86.96%, respectively (17). Whereas, Elhosseiny et al. found these values to be 65.2% and 100%, respectively (18). We have found the sensitivity and specificity of Alvarado scores to be 60.9% and 89.9%, respectively. Khan et al. reported NA and perforated appendectomy rates to be 15.6% and 7.8%, respectively (19). Researchers have been trying to develop better diagnostic methods to decrease these numbers.

Studies suggest that the RIPASA score is more accurate than the Alvarado score, especially in Eastern societies (18). Frountzas et al. studied 2161 cases of AA and found that while the RIPASA system was more sensitive, it had a lower specificity than the Alvarado system (20). Chong et al. studied the RIPASA scoring system, and found that it had 97.5% sensitivity, 81.8% specificity and 91.8% diagnostic accuracy (21). We have found that the AUC for the RIPASA score was slightly lower than the Alvarado score (0.893 vs. 0938).

The Ohmann score is a simple test that can help diagnose patients with suspected AA (22). Similarly, the Eskelinen score is considerably successful in ruling out the diagnosis of AA (23). Erdem et al. found that the sensitivity and specificity of the Ohmann and Eskelinen scores 96% and 42%, and 100% and 44%, respectively (24). We found that Ohmann and Eskelinen scores failed to diagnose AA, but they were sufficiently specific. The Eskelinen score is at a disadvantage due to its decimal calculations that make it less practical. It also may require additional diagnostic methods, such as laboratory testing or ultrasonography, for differential diagnosis. 

The Tzanakis score was suggested as a combined clinical evaluation of US results and inflammatory markers, the highest possible score is 15, and ≥8 indicates AA. The sensitivity and specificity were 95.4% and 97.4%, respectively (25). Sigdel et al. reported that the Tzanakis score was as effective as the Alvarado score, with a lower false-negative rate (26). Studies show sensitivity levels to be between 85-96%, but Sigdel et al. attribute these low rates to differences in the experience levels of radiologists that perform US (26, 27). 

## Limitation

The limitations of our study are as follows: (a) the relatively small sample size despite the prospective nature of the study, and (b) different physicians deciding for appendectomy for different cases. Further prospective studies with larger sample sizes are required to support our findings. 

## Conclusion

Tzanakis score has higher sensitivity and specificity in diagnosis of AA compared to Alvarado, RIPASA, Eskelinen and Ohmann scores. 
